# The mycoremediation potential of the armillarioids: a comparative genomics analysis

**DOI:** 10.3389/fbioe.2023.1189640

**Published:** 2023-08-17

**Authors:** Simang Champramary, Boris Indic, Attila Szűcs, Chetna Tyagi, Omar Languar, K. M. Faridul Hasan, András Szekeres, Csaba Vágvölgyi, László Kredics, György Sipos

**Affiliations:** ^1^ Functional Genomics and Bioinformatics Group, Institute of Forest and Natural Resource Management, Faculty of Forestry, University of Sopron, Sopron, Hungary; ^2^ Department of Microbiology, Faculty of Science and Informatics, University of Szeged, Szeged, Hungary; ^3^ Fibre and Nanotechnology Program, Faculty of Wood Engineering and Creative Industries, University of Sopron, Sopron, Hungary

**Keywords:** mycoremediation, biodegradation, armillarioids, white-rot, phylogenetic principal component analysis, benzoate 4-monooxygenase

## Abstract

Genes involved in mycoremediation were identified by comparative genomics analysis in 10 armillarioid species and selected groups of white-rot Basidiomycota (14) and soft-rot Ascomycota (12) species to confine the distinctive bioremediation capabilities of the armillarioids. The genomes were explored using phylogenetic principal component analysis (pPCA), searching for genes already documented in a biocatalysis/biodegradation database. The results underlined a distinct, increased potential of aromatics-degrading genes/enzymes in armillarioids, with particular emphasis on a high copy number and diverse spectrum of benzoate 4-monooxygenase [EC:1.14.14.92] homologs. In addition, other enzymes involved in the degradation of various monocyclic aromatics were more abundant in the armillarioids than in the other white-rot basidiomycetes, and enzymes involved in the degradation of polycyclic aromatic hydrocarbons (PAHs) were more prevailing in armillarioids and other white-rot species than in soft-rot Ascomycetes. Transcriptome profiling of *A. ostoyae* and *A. borealis* isolates confirmed that several genes involved in the degradation of benzoates and other monocyclic aromatics were distinctively expressed in the wood-invading fungal mycelia. Data were consistent with armillarioid species offering a more powerful potential in degrading aromatics. Our results provide a reliable, practical solution for screening the likely fungal candidates for their full biodegradation potential, applicability, and possible specialization based on their genomics data.

## 1 Introduction

Polycyclic aromatic hydrocarbons (PAHs), chemical dyes, plastics, heavy metals, and pharmaceutical waste, all of which are distinct chemical wastes, pose a significant threat to all life forms on Earth and therefore require the development of environmentally friendly, cost-effective, and efficient solutions to combat them (Barh et al., 2019). Even though strides have been made to combat these issues using traditional chemical remediation methods, namely, precipitation, reverse osmosis, and reduction, they have often proved inefficient and too costly (Kapahi and Sachdeva, 2017). However, various bioremediation procedures like myco- and phytoremediation, biostimulation, bioaugmentation, and composting have emerged as potential alternatives to chemical-based interventions by offering greater flexibility and cost-effectiveness (Taiwo, 2011; Tyagi et al., 2011; Rhodes, 2014; Herrero and Stuckey, 2015; Ali et al., 2017; Ansari et al., 2020; Yadav et al., 2021).

Mycoremediation, as one of these potential alternatives, represents the degradation of pollutants in the soil or water with the aid of fungi and their enzymes (Barr and Aust, 1994). The two main activities of the mycoremediation processes are biodegradation and biosorption (Kulshreshtha et al., 2014). In biodegradation, the fungus uses its enzymes to break down the pollutants into less complex and non- or less-toxic products. Biosorption is the process by which fungi become tolerant to specific toxic contaminants, *e.g*., heavy metals, accumulate them, and then remove such impurities from the soil by continuous sorption. Finally, bioconversion or biotransformation refers to the utilization of organic waste by fungi to generate energy and support their growth.

Lignin-degrading white-rot fungi from Basidiomycota, including *Phanerochaete chrysosporium*, *Pleurotus ostreatus*, *Irpex lacteus*, and *Schizopora* spp., and also certain Ascomycota species like *Botryosphaeria* spp. and *Aspergillus niger*, utilize enzymes, such as laccases, cytochrome p450 monooxygenases, FAD monooxygenases, cutinases, peroxidases, hydrolases, and antioxidants to cope with toxic pollutants like heavy metals, PAHs, and fuel oils (Akhtar and Mannan, 2020). Due to their lignin-degrading enzymes including lignin peroxidases (LiP), manganese peroxidases (MnP), and laccases (Lac), white-rot fungi primarily have an extracellularly exposed oxidative potential which can target both phenolic and non-phenolic substances in their environment. LiPs act on non-phenolic compounds, whereas MnPs and Lacs act on phenolic ones (McMullan et al., 2001).

Recently, the mechanisms of lignin degradation by peroxidases, laccases, and glutathione-dependent β-etherase enzymes were comprehensively studied by Cajnko et al. (2021). Peroxidases, known as monomeric heme-containing enzymes, depolymerize lignin through a three-step oxidation process, whereas Lacs–which are versatile low substrate specificity multicopper oxidases–catalyze the oxidation of various aromatic substrates using copper ions. A further emerging alternative lignin depolymerization technique is based on the β-etherase system, which selectively catalyzes the reductive cleavage of lignin’s β-O-4 aryl-ether bonds. Concerning the complexity of degrading lignin and PAHs, Cajnko et al. (2021) also disclosed the importance of controlling the simultaneous depolymerization and polymerization trends of aromatics during lignin degradation and that the competition between depolymerization and polymerization could be affected by several variables, including pH, temperature, enzyme/substrate ratio, enzyme concentration, and incubation time. Most importantly, the efficiency of the depolymerization process was also influenced by the kinetic parameters of various enzymes, and beyond that, it was also observed that the same type of enzyme from a different fungal source, or another isoform of the same enzyme from the same source, would have significantly altered affinities for the same substrates (Patel et al., 2014; Surendran et al., 2018).

When considering a fungal resource in a bioremediation intervention, multilevel tasks must be evaluated and fulfilled, starting from selecting the appropriate fungal isolates and then moving toward optimizing the most suitable technological solutions and engineering conditions.

Regarding the practical biotechnological implementations when employing white-rot fungi, Babič et al. (2012) used response surface methodology (RSM) to demonstrate the activity and productivity of the ligninolytic MnP and Lac enzymes produced by the white rot fungus *Ceriporiopsis subvermispora* in submerged cultures. They observed that increasing Lac activities required a two-step approach, including liquid media optimization and appropriate immobilization support, the latter benefitting from the innate ability of filamentous fungi to adhere to surfaces and boost Lac activities.

At the level of bioengeneering, Kogej et al. (2010) studied the lead (Pb) biosorption potential of *Rhizopus nigricans* using an experimental packed bed reactor and modeled the biosorption process mathematically. The model was numerically solved and found to be in good agreement with the experimental data. A parameter sensitivity study revealed that the model was sensitive to biomass properties and various operating conditions, including liquid flow rate, and initial Pb concentration.

Glenn and Gold (1983) first observed the involvement of white-rot species in the breakdown of aromatic dyes and have since triggered a flurry of research. Recently, Yesilada et al. (2018) and Rajhans et al. (2021) have reviewed these topics comprehensively. Fungal oxidation affects all dyes, but there are significant differences between fungal species regarding their catalytic performance and dye oxidation potential. On the other hand, the biodegradation chemical pathways fungi use are also inconsistent in treating certain dyes assuming rather structure-dependent degradation mechanisms (Alam et al., 2023; Gul et al., 2023). However, so far, no obvious link has been found between the structure of a dye and its biodegradability by fungi (Fu and Viraraghavan, 2001).

When nutrient resources (mainly C, N, and S) become scarce, fungi degrade aromatic structures as a secondary metabolic event (Rabinovich et al., 2004). Earlier, researchers used biochemical assays to study the impact of enzymes on various pollutants in the presence of fungi. Current studies focus on using an *in vitro* fungal culture technique to investigate the mycoremediation potential of a limited number of fungal species isolated from the contaminated sites or previously reported to be involved in specific mycoremediation activities (Lee et al., 2014; Tkavc et al., 2018). Although such an approach is scientifically sound, there is a gap as the fungal species chosen for the experiments may not be the most effective in the bioremediation of the specific pollutants (Matsubara et al., 2005).

Lately, with advances in next-generation sequencing, we can better predict and understand the bioremediation potential of a fungus by using a comparative genomics approach. Genome-level comparisons can reveal significant changes in metabolic pathways and project the physiological capacity of fungi for mycoremediation (Dao et al., 2019; Park and Choi, 2020). This approach has been substantiated by Park et al. (2019), who used comparative genomics and transcriptomics analysis to study the PAH degradation potential of the white rot fungus *Dentipellis* sp. KUC8613, which was previously reported by Lee et al. (2014). In their study, they identified the enzymes that were used for degrading PAHs. The genes upregulated during the stage of PAH degradation were P450s, epoxide hydrolases, alcohol/aldehyde dehydrogenases, monooxygenases, and dioxygenases. Also, while studying the DDT-resistant *Trichoderma hamatum* FBL 587, Davolos et al. (2021) observed that the fungi had increased repertoires of xenobiotic-degrading enzymes and specialized DNA-repairing mechanisms. Additionally, Krijger et al. (2014) observed that the ability of a fungus to grow and survive in a complex, challenging environment is directly correlated with the expansion of certain genes/protein families. Similarly, Muszewska et al. (2017) identified that specific families of serine peptidases like S1, S8, S41, S54, S64, and S66 correlated with the fungal lifestyle. These studies indicate that screening for the xenobiotic-degrading enzyme repertoires in different fungal species can help us identify the best fungal candidates and make informed decisions when choosing a specific fungal species for particular pollutants.

Taking all of the above into consideration, we utilized a recently established database of biodegradative enzymes to compare the putative bioremediation-related gene sets of the armillarioid species (Kedves et al., 2021) with those of 14 white-rot Basidiomycota and 12 Ascomycota fungi (Supplementary File 1; Supplementary Figure 1) and by doing so, based on their gene/enzyme profiles, to identify which armillarioid species would have the highest potential for use in the mycoremediation process. Our comparative analysis included Ascomycota species due to recent findings that, besides lignin-degrading activities, an ancestral soft-rot machinery was also shared across Asco- and Basidiomycota (Sahu et al., 2021).

## 2 Materials and methods

### 2.1 Genomic datasets

Amino acid sequences of 7 *Armillaria* species (*A. borealis* (Armbor1), *A. cepistipes* B5 (Armcep1), *A. gallica* 21-2 v1.0 (Armga1), *A. luteobubalina* HWK02 v1.0 (Armlut1), *A. mellea* (Armmel1), *A. ostoyae* C18/9 (Armosto1), *A. solidipes* 28-4 v1.0 (Armost1)), 2 *Desarmillaria* species (*D. tabescens* CCBAS 213 v1.0 (Armtab1), *D. ectypa* FPL83.16 v1.0 (Armect1)), *Guyanagaster necrorhizus* MCA 3950 v1.0 (Guyne1), 14 white-rot basidiomycetous fungi (*Heterobasidion annosum* v2.0 (Hetan2), *Polyporus squamosus* CCBS 676 v1.0 (Polsqu1), *Schizophyllum commune* H4-8 v3.0 (Schco3), *Bjerkandera adusta* v1.0 (Bjead1_1), *I. lacteus* CCBAS Fr. 238 617/93 v1.0 (Irplac1), *Lentinula edodes* Le (Bin) 0899 ss11 v1.0 (Lenedo1), *Marasmius fiardii* PR-910 v1.0 (Marfi1), *P. chrysosporium* RP-78 v2.2 (Phchr2), *Phlebia radiata* Fr. (isolate 79, FBCC0043) (Phlrad1), *Pleurotus eryngii* ATCC 90797 v1.0 (Pleery1), *P. ostreatus* PC9 v1.0 (PleosPC9_1), *Trametes betulina* CIRM-BRFM 1801 v1.0 (Trabet1), *Trametes versicolor* v1.0 (Trave1), *Dentipellis* sp. KUC8613 (Densp1), and 12 ascomycetes (*Trichoderma citrinoviride* TUCIM 6016 v4.0 (Trici4), *Trichoderma harzianum* TR274 v1.0 (Trihar1), *Aspergillus flavus* NRRL3357 (Aspfl2_3), *Aspergillus glaucus* v1.0 (Aspgl1), *Botryosphaeria dothidea* (Botdo1_1), *Cladosporium sphaerospermum* UM 843 (Clasph1), *Cochliobolus lunatus* m118 v2.0 (Coclu2), *Fusarium oxysporum* Fo5176 (FoxFo5176), *Galactomyces geotrichum* Phaff 72-186 (Galgeo1), *Penicillium arizonense* CBS 141311 (Penar1), *Penicillium chrysogenum* Wisconsin 54-1255 (PenchWisc1_1), *Purpureocillium lilacinum* PLFJ-1 (Purli1), and 1 mucormycete (*Mucor circinelloides f. lusitanicus* MU402 v1.0 (Muccir1_3)) as an outlier species were downloaded from JGI (https://mycocosm.jgi.doe.gov/mycocosm/home) (Supplementary File 1).

### 2.2 *In vitro* stem invasion assays

Gene expression data from recently published *in vitro* stem invasion assays (Sahu et al., 2023) were further analysed to investigate the expression of mycoremediation-related genes in *Armillaria* isolates growing on RSTO media (Sipos et al., 2017), which provides an artificially rich nutrient source, and in mycelia invading and growing in plant tissues. In the stem invasion assays, fresh spruce stem segments were placed on the mycelial lawn grown on RSTO media. Gene expression profiles of highly virulent and less virulent isolates of two conifer-specific *Armillaria* species (*A. borealis* and *A. ostoyae*) were examined for the expression of mycoremediation genes. All further experimental details, analysis of the RNA-Seq data, and identification of the gene expression levels of the genes of interest are described in Sahu et al. (2023).

### 2.3 Data analysis

KofamScan (Aramaki et al., 2020), which employs a hidden Markov model for identifying the KEGG (Kyoto Encyclopedia of Genes and Genomes) orthology of the sequences, was then used for functional annotation of amino acid sequences. KofamScan was run by applying the default parameters, and the significant annotations were selected based on the KofamScan’s adaptive score thresholds and marked with “*.” KEGG enzyme IDs were then used to curate the significant hits using the Biocatalysis/Biodegradation Database (http://eawag-bbd.ethz.ch/) (Gao et al., 2010). The remaining data which could not be curated using the Biocatalysis/Biodegradation Database were manually curated using the KEGG database (Kanehisa et al., 2016). Finally, the enzymes involved in xenobiotic degradation from the KEGG database were considered for comparison using R v4.2 for computation and calculations.

Phylogenetic principal component analysis (pPCA) was performed using the R packages adenophylo v1.1-11 (Jombart et al., 2010). The enzyme copy numbers were counted for each fungus and converted into the n x m matrix to create the pPCA. The phylogenetic tree (Supplementary Figure 1) used in the pPCA analysis was constructed using single copy orthologs. MAFFT v7 aligner (Katoh and Standley, 2013) was applied for sequence alignment, and FastTree v2.1 (Price et al., 2010) was operated to generate the maximum-likelihood phylogenetic trees using the aligned sequences. All images were plotted applying the R package ggplot2 (Wickham, 2016). Multiple sequence alignment of amino acid sequences was performed using MEGAx (Stecher et al., 2020), and motif analysis was completed using the MEME Suite (Bailey et al., 2015). Heme and substrate binding motifs were predicted using the NCBI Conserved Domain Database (Marchler-Bauer et al., 2015). The phylogenetic trees, incorporating structural motifs and/or expression data, were visualized using the Interactive Tree of Life (iTol) tool (Letunic and Bork, 2021).

## 3 Results

### 3.1 Comparative analysis of genes potentially involved in biodegradation

**FIGURE 1:**
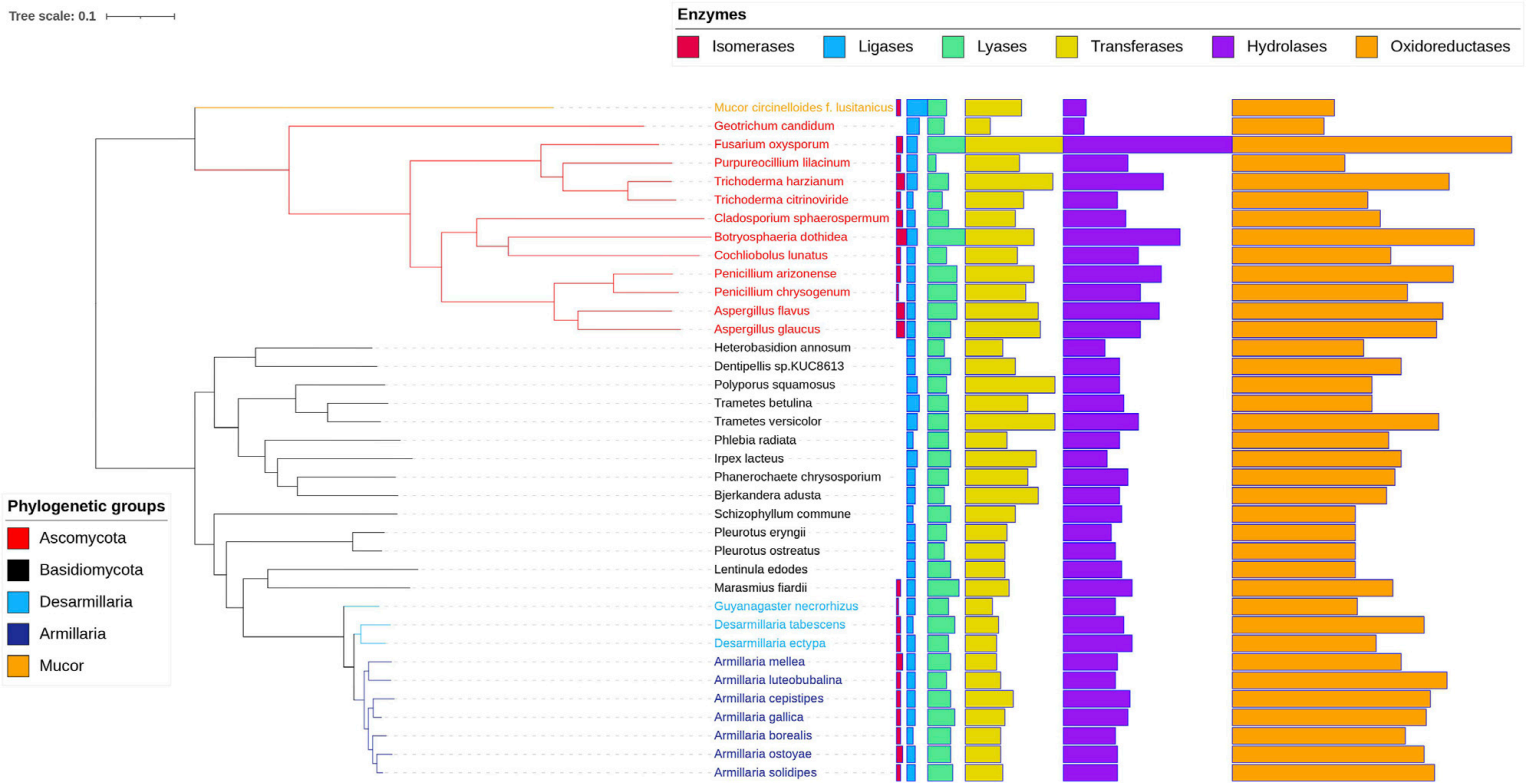
Diagrams of the KEGG enzyme classes of the fungi used in the study. The top X-axis lists the enzyme classes, while the Y-axis lists the names of the fungi. The maximum likelihood phylogenetic tree of fungal species was constracted using orthologous proteins.

To identify genes involved in mycoremediation in various white-rot specific fungi, 10 armillarioids (seven *Armillaria*, two *Desarmillaria*, and one *Guyanagaster*), 14 white-rot Basidiomycota combined with 12 Ascomycota and one Mucoromycota as outlier species were considered for the genome-level comparative analysis (Supplementary File 1). We first performed KofamScan on 536,770 amino acid sequences from the 37 selected fungal genomes and found 159,088 unique fungal proteins that could be significantly associated with KEGG functions. Then, following manual curation, 5,526 protein sequences related to biodegradation could be identified. The gene repertoire patterns of the key enzyme classes were consistent in all fungal genomes except for five basidiomycetous (*B. adusta*, *T. betulina*, *T. versicolor*, *P. squamosus*, *I. lacteus*) and two ascomycetous (*T. citrinoviride*, *G. candidum*) species, and in *M. circinelloides* f. *lusitanicus*, where the total number of transferase enzymes was higher than the total number of hydrolases (Figure 1; Supplementary File 1).

**FIGURE 2:**
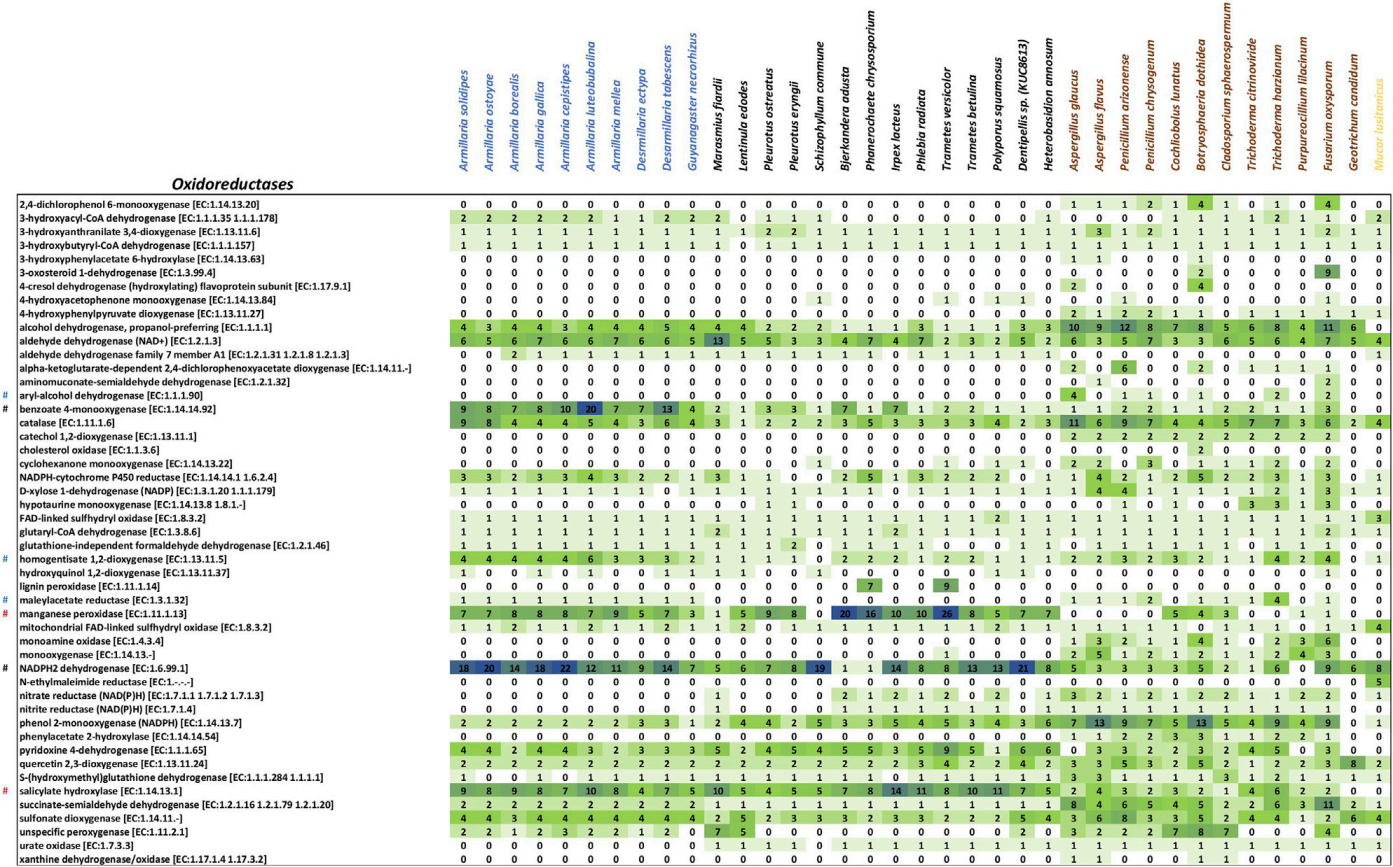
Heatmap of mycoremediation-related oxidoreductase counts. Fungal species are on the top X-axis, and the enzyme names are listed along the Y-axis. The intensity of the cell color correlates with the number of counts of a particular enzyme. # Enzyme clusters enriched in Armillarioids. # Involved in PAH degradation. # Involved in degrading monocyclic aromatics.

**FIGURE 3:**
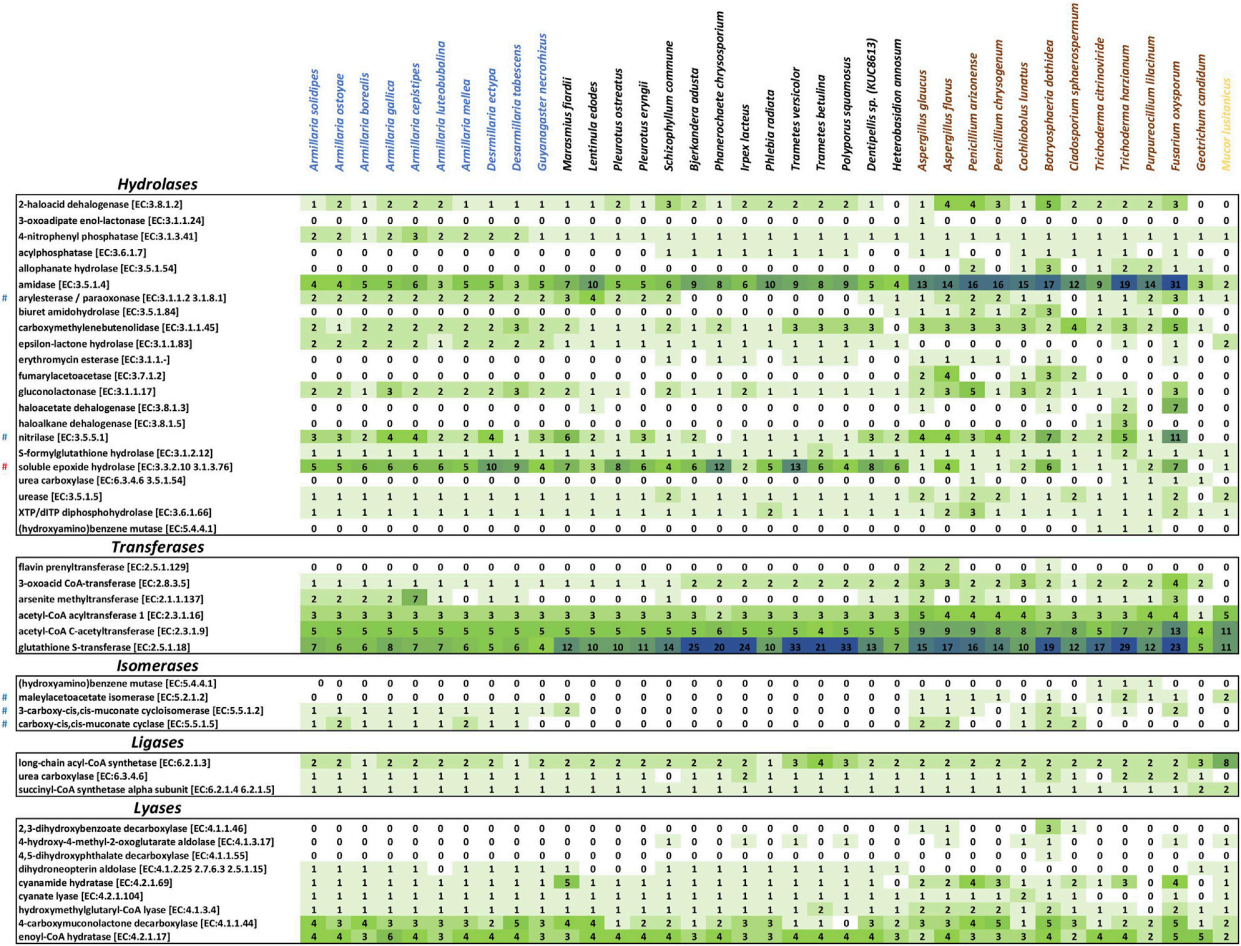
Heat maps of hydrolase, transferase, isomerase, ligase and lyase counts associated with mycoremediation. Fungal species are at the top of the X-axis, and the enzyme names are along the Y-axis. The intensity of the cell color correlates with the number of counts of a particular enzyme. # Involved in PAH degradation. # Involved in degrading monocyclic aromatics.

When assessing the possible specialization within white-rot species, the copy numbers of 92 unique enzymes representing a broad spectrum of biodegradative activities (Figures 2, 3; Supplementary File 1) were analyzed and compared in all selected genomes. pPCA of the prospective biodegradation genes demonstrated that armillarioid species clustered together and separated well from the other white-rot species (Supplementary Figures 2, 3). Notably, based on the individual copy number profiles, the genes coding for benzoate-4-monooxygenase [EC:1.14.14.92] and NADPH_2_ dehydrogenase [EC:1.6.99.1] (or xenobiotic reductase, "EAWAG-BBD enzyme, enzymeID# e0038″) homologs, both cases with exclusively high copy numbers in the armillarioid genomes appeared to have significantly contributed to their separate clustering (Figure 2).

Phylogenetic analysis of 143 benzoate-4-monooxygenase and 331 NADPH_2_ dehydrogenase proteins from all fungi uncovered a diverse family of both proteins (Supplementary Figures 4, 5). Despite the apparent structural diversity, no significant substitutions were observed in the armillarioid NADPH_2_ dehydrogenase proteins compared to Ascomycota and Basidiomycota based on their MEME motif analysis of the active site, substrate-binding, and cofactor-binding sites (data not shown). However, multiple sequence alignment analysis revealed slight but significant differences in the substrate- and heme-binding sites of the benzoate-4-monooxygenases (Supplementary Figure 6). The variations in hydrophobicity and the size of substitutions at the 7th and 2nd positions of the substrate-binding site may affect the overall shape of the substrate-binding surface, thereby increasing or descreasing specificity. Furthermore, at the 6th position of the heme-binding site, a hydrophobic leucine in armillarioids is replaced with a polar, hydrophilic glutamin in Basidiomycota and Ascomycota. It is known that a more hydrophobic environment is preferred in the interaction of heme with the binding site (Liou et al., 2014). These possibilities require further extensive experimentation to validate.

### 3.2 Genes involved in degrading monocyclic aromatics

In general, genes other than benzoate 4-monooxygenase involved in modifying and degrading various monocyclic aromatic compounds, namely, homogentisate 1,2-dioxygenase (HGD) [EC:1.13.11.5] (Figure 2), aryl-alcohol dehydrogenase [EC:1.1.1.90] (Figure 2), maleylacetate reductase [EC:1.3.1.32] (Figure 2), arylesterase/paraoxonase [EC:3.1.1.2/3.1.8.1] (Figure 3), nitrilase [EC:3.5.5.1] (Figure 3), maleylacetoacetate isomerase [EC:5.2.1.2] (Figure 3), and 3-carboxy-cis,cis-muconate cycloisomerase [EC:5.5.1.2] (Figure 3) were randomly distributed in the genomes analyzed. HGD, maleylacetate reductase, and nitrilase genes were relatively more prevalent in armillarioids and ascomycetes (Figures 2, 3; Supplementary File 1).

Notably, the gene copy numbers of HGDs were predominantly higher in all the armillarioid species and certain ascomycetous white-rot species than in other basidiomycetes. *A. luteobubalina* had 6 copies of HGD genes, and other *Armillaria* species had at least 3 copies. From the ascomycetous genomes, *T. harzianum* and *F. oxysporum* contained the highest copy number of HGDs (4 copies each).

Maleylacetate reductase [EC:1.3.1.32]—converting various aromatic compounds (resorcinol, fluorobenzoate, chlorocyclohexane, chlorobenzene, toluene, benzoate) to 3-oxoadipate–was present as a single copy in all armillarioid species but was missing from the genomes of all other basidiomycetes. The copy number distribution of maleylacetate reductases was not homogenous in ascomycetes as some had higher copies, and some did not show a single copy of the gene. For example, *T. harzianum* and *P. chrysogenum* manifested 4 and 2 copies of maleylacetate reductases, respectively.

Similarly to maleylacetate reductase genes, all *Armillaria* spp., *Desarmillaria* spp., and some ascomycetous fungi shared comparable copy numbers of 3-carboxy-cis,cis-muconate cyclase [EC:5.5.1.5] (Figure 3) genes. Regarding nitrilases acting potentially also on certain aromatic compounds, armillarioids and ascomycetous fungi had average copy numbers of 2 and 3 nitrilases [EC:3.5.5.1], respectively, while other basidiomycetous fungi had a mean copy of only one. The copy number of arylesterase/paraoxonase (Figure 3), an aminobenzoate-degrading enzyme, was comparatively higher in armillarioids than in other basidiomycetes and ascomycetes.

Other genes encoding aromatic-degrading enzymes, aryl-alcohol dehydrogenases, and maleylacetoacetate isomerases (Figures 2, 3) occur only in ascomycetous species.

### 3.3 Genes responsible for degrading PAHs

Gene copy numbers of salicylate hydroxylases [EC:1.14.13.1] (Figure 2), soluble epoxide hydrolases [EC:3.3.2.10] (Figure 3) and MnPs [EC:1.11.1.13] (Figure 2), which partake in the breakdown of PAHs were much higher in basidiomycetous fungi. More copies (≥4) of soluble epoxide hydrolases [EC:3.3.2.10] (EAWAG-BBD reaction, reacID# r1120), which are benzo [a]pyrene (PAH) -degrading enzymes (Keck et al., 2006), have their genes in armillarioids and the other white-rot basidiomycetes. One or two copies of the gene were present in the ascomycetous fungi except for *F. oxysporum* and *B. dothidea*, which possessed 7 and 6 copies, respectively.

The mean gene copy number of another PAH-degrading enzyme, salicylate hydroxylase [EC:1.14.13.1] (EAWAG-BBD enzyme, enzyme ID: e0149) (Figure 2) was also considerably much higher in basidiomycetes compared to ascomycetes. The basidiomycetous species *A. luteobubalina*, *A. mellea*, *A. ostoyae*, *A. solidipes*, *I. lacteus*, *M. fiardii*, *P. chrysosporium*, *P. radiata*, *P. squamosus*, *T. betulina*, and *T. versicolor* had ≥8 copies of salicylate hydroxylase genes, whereas in ascomycetes the average copy number was just 2.

The distribution of the MnP genes [EC:1.11.1.13] (Figure 2) was homogenous (8 copies on average) in all basidiomycetes except in *B. adusta* (20 copies), *T. versicolor* (26 copies) and *P. chrysosporium* (16 copies), the ones which showed an extremely high number of anthracene-degrading MnPs. *C. lunatus*, an ascomycetous fungus, had 5 copies of MnPs, which was the highest among other ascomycetes comprising only 1 or 2 copies.

### 3.4 Arsenite methyltransferase, epsilon-lacton hydrolase, and sulfonate dioxygenase enzymes for the bioremediation of antibiotics, caprolactam, and sulphonate-based herbicides

Arsenite methyltransferase [EC:2.1.1.137] (Figure 3), involved in the first conversion step of organoarsenic compounds such as methylarsonic acid to dimethylarsinate (EAWAG-BBD reaction, reacID: r0805) (Shah and Damare, 2018), was found to be most abundant in *A. cepistipes*, which had an exclusive 7 copies while *A. solidipes*, *A. ostoyae*, *A. borealis*, and *A. gallica* had only 2 copies each. In addition, 1 copy of the arsenite methyltransferase gene was found in *A. luteobubalina*, *D. tabescens*, *D. ectypa*, *S. commune*, *Dentipellis* sp. KUC8613, and *H. annosum,* but not in *A. mellea* or *G. necrorhizus*. On average, ascomycetous white-rot fungi possessed one copy of arsenite methyltransferase, and among all studied ascomycetous fungi, *F. oxysporum* had the highest number (3 copies).

All armillarioid species except *A. luteobubalina* possessed 2 copies of caprolactam-degrading epsilon-lactone hydrolases [EC:3.1.1.83] (Figure 3). *A. luteobubalina* and other basidiomycetes had only 1 copy of epsilon-lactone hydrolase. They were also found to be almost non-existent in the ascomycetous white-rot species.

The gene copy number of sulfonate dioxygenase [EC:1.14.11.-] (Figure 2), a sulfonate-degrading enzyme, was higher in ascomycetous fungi and armillarioid species than in other basidiomycetes. Their number was evenly distributed in *Armillaria* but varied from 1 to 8 among ascomycetous fungi. Two basidiomycetous species, *L. edodes* and *Dentipellis* sp. KUC8613 were exceptions as their genomes contained 5 copies.

### 3.5 RNA-seq profiling of genes involved in the degradation of various aromatic compounds

Initial expression profiling of genes with mycoremediation potential was performed using raw RNA-Seq data from recently published *in vitro* stem invasion assays (Sahu et al., 2023) to analyse their expression under native plant-interactive and artificial, rich media conditions. The RSTO medium, which provides a broad spectrum of substrates, is essential for *Armillaria* species initiating fruiting body formation and a critical inoculum allowing the invasiveness of less virulent isolates in stem invasion experiments. Gene expression profiles were explored in single pairs of highly virulent and less virulent isolates of two conifer specific *Armillaria* species (*A. borealis* and *A. ostoyae*) and found that the majority of identified mycoremediation genes were expressed in both species, 95% in *A. ostoyae* and 87% in *A. borealis* (Supplementary File 1; Table 1).

**TABLE 1:** *In vitro* stem invasion assays–Expression analysis of the mycoremediation-related genes.

	*A. ostoyae* (expressed/total in genome)	*A. borealis* (expressed/total in genome)
Mycoremediation related genes	130/137 (95%)	126/145 (87%)
Benzoate degradation		
Benzoate-4-monooxygenase [EC:1.11.1.13]	6/8	7/7
Monocyclic aromatics degraders	9/12 (75%)	10/11 (91%)
Homogentisate 1,2-dioxygenase [EC:1.13.11.5]	4/4	4/4
Maleylacetate reductase [EC:1.3.1.32]	1/1	1/1
Arylesterase/paraoxonase [EC:3.1.1.2 3.1.8.1]	2/2	2/2
Nitrilase [EC:3.5.5.1]	3/3	2/2
3-carboxy-cis,cis-muconate cycloisomerase [EC:5.5.1.2]	0/1	1/1
Carboxy-cis,cis-muconate cyclase [EC:5.5.1.5]	0/2	0/1
PAH degraders	15/20 (75%)	21/23 (91%)
Salicylate hydroxylase [EC:1.14.13.1]	7/8	9/9
Manganese peroxidase [EC:1.11.1.13]	4/7	7/8
Soluble epoxide hydrolase/lipid-phosphate phosphatase [EC:3.3.2.10 3.1.3.76]	4/5	5/6

The table compares the expressed genes detected in the mycelia growing under native plant interactive or artificial reach media conditions with the total number of related genes identified in the *A. ostoyae* and *A. borealis* genomes. Genes were expressed if their CPM (counts per million) value was higher than 1 CPM in at least two biological samples. All biological sample data represent the mean values of three biological replicates.

**FIGURE 4:**
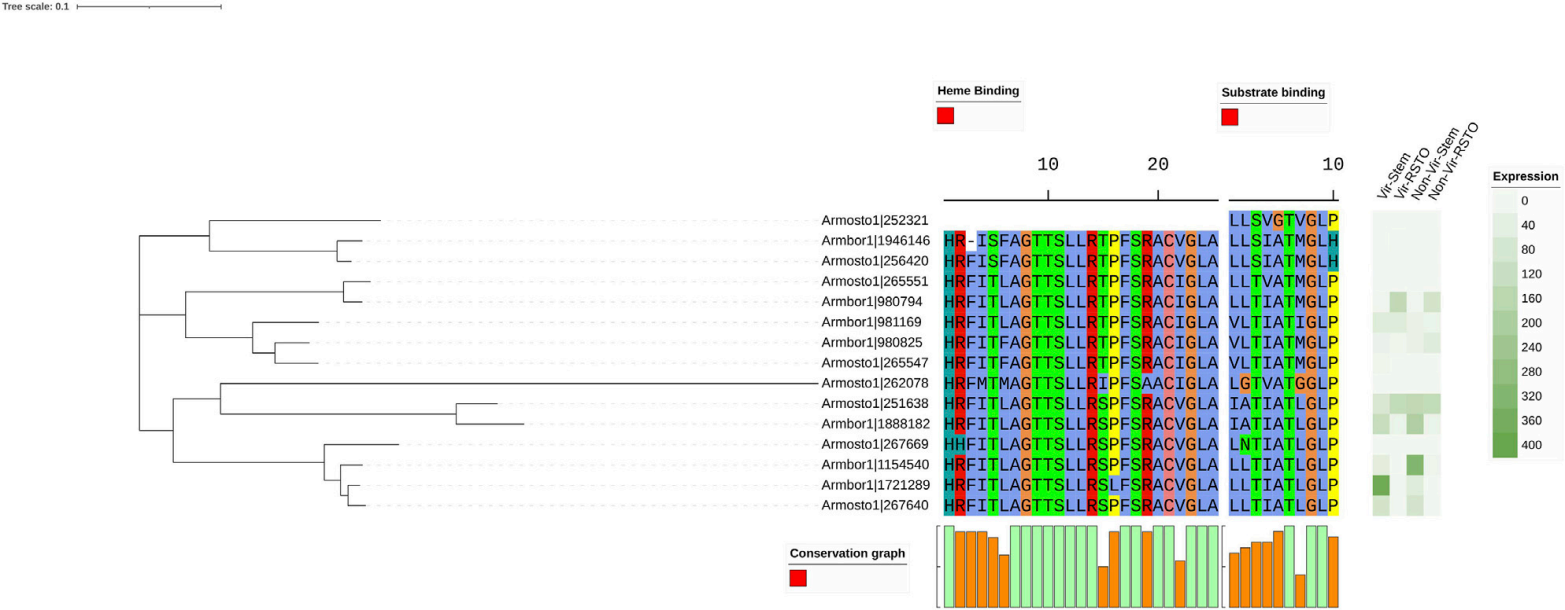
Heatmaps of gene expression and alignments of heme and substrate binding sites of the benzoate-4-monooxygenase genes. High and low virulent *A. ostoyae* and *A. borealis* isolate pairs were used in the *in vitro* stem invasion assays. “Vir” and “Non-Vir” refer to the high and low virulent isolates, respectively, and “Stem” and “RSTO” indicate mycelia isolated from the underbark tissue of stem segments and mycelia growing on rich media.

For the comparative expression analysis of the benzoate-4-monooxygenase genes, the individual substrate and heme-binding sites of the genes were also included (Figure 4). Interestingly, 4 out of 5 genes having leucine or alanine at the 2nd and leucine at the 7th position in the substrate-binding sites–representing the most likely armillarioid-specific residues (Supplementary Figure 6)—showed an *in planta* response where genes were upregulated under stem-invasive conditions in all isolates of both species. Notably, 1 *A. borealis* gene (Ambor|1721289) appeared to contribute to virulence, as it was significantly overexpressed under plant-invasive conditions in the virulent isolate compared to the less-virulent one. In contrast, 7 other genes with altered substitutions at the 2nd and/or 7th positions, or significantly different replacements at other positions in the binding site were silent.

**FIGURE 5:**
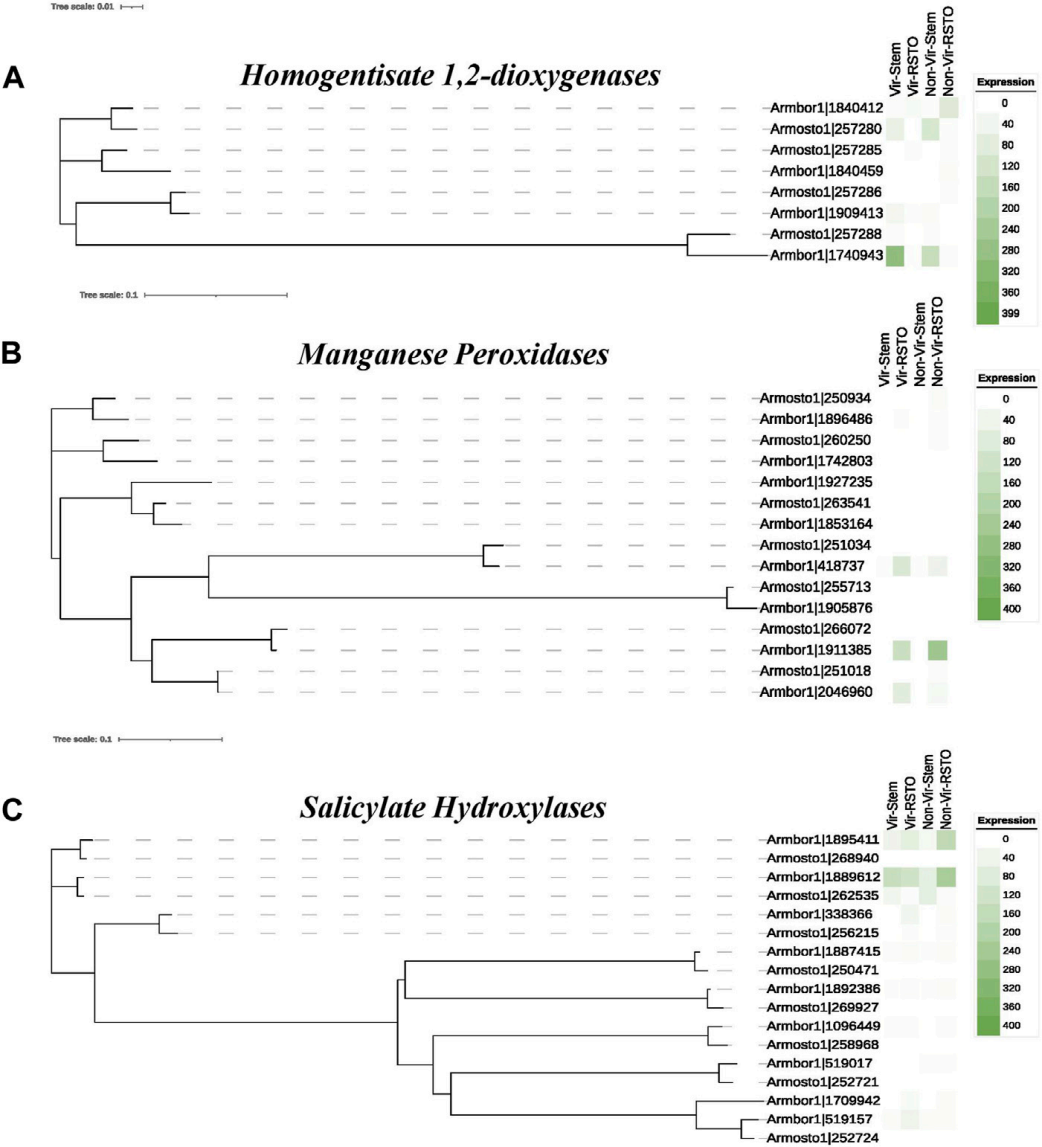
Heatmaps of the expression of HGD **(A)**, MnP **(B)** and salicylate-hydroxylase **(C)** genes. High and low virulent *A. ostoyae* and *A. borealis* isolate pairs were used in the *in vitro* stem invasion assays. “Vir” and “Non-Vir” refer to the high and low virulent isolates, respectively, and “Stem” and “RSTO” indicate mycelia isolated from the underbark tissue of stem segments and mycelia growing on rich media.

The homogentisate 1,2-dioxygenase genes were also relatively more abundant in *Armillaria* species. Their comparative gene expression profiling showed that 3 of the 8 genes, similarly to benzoate-4-monooxygenases, were also upregulated under plant-invasive conditions, while the rest was silent or expressed differently (Figure 5).

Regarding the gene expression profiles of salicylate hydroxylases and MnPs, both involved in the degradation of PAHs and prevalent in Basidiomycota, they showed different expression patterns from benzoate monooxygenase and HGD genes (Figure 5) as their expression levels tended to increase on rich media.

## 4 Discussion

In the search for ecologically friendly and effective practical solutions to environmental pollution, mycoremediation proves to be a promising option that meets all the necessary criteria, such as cost efficiency, environmental friendliness, and effectiveness. Additionally, using fungi and fungal enzymes to treat various chemical wastes can offer greater flexibility and cost-effectiveness than typical chemical remediation techniques.

Our preliminary tests using an initial comparative genomics analysis to predict mycoremediation-related genes in *Dentopellis* sp. (KUC8613) identified 141 genes in the *Dentopellis* genome. Among these genes, 34 were significantly upregulated in a PAH-based mycoremediation experiment conducted by Park et al. (2019). The strong positive correlation (R = 0.86, *p*-value = 2.3e-07) observed for the upregulated enzyme repertoire within the expressed genome level counts suggested that the differential expression of these genes may indicate the genetic potential within the genomes and also implied that the presence and abundance of mycoremediation-related genes in a genome can be linked to enzymatic activities and capabilities required for efficient biodegradation. These findings prompted us to explore further and compare the gene repertoires in other fungal species, focusing on possible functional diversities and specializations within expanded bioremediation-related functions.

An extensive comparative genomics analysis of xenobiotics-degrading gene/protein copy numbers in 36 fungal species gave us more profound insights into the distribution and abundance of key enzymes involved in mycoremediation across diverse taxa. Our findings revealed that armillarioid species have a distinctive potential to degrade benzoic acid derivatives. It was reflected in the genetically diverse, increased repertoires of benzoate-degrading benzoate 4-monooxygenase genes in the armillarioid species (Supplementary Figure 4). Consequently, these data suggest that armillarioid species could efficiently treat environmental benzoate contaminations. However, benzoate 4-monooxygenase [EC 1.14.14.92], an oxidoreductase enzyme in high copy numbers in the genomes of armillarioids, offers only a single step in transforming benzoate to 4-hydroxybenzoate (Fuchs et al., 2011). The genes involved in further converting 4-hydroxybenzoate still need to be identified, and the complete degradation process should be adequately studied.

Based on the increased repertoire of NADPH_2_ dehydrogenase/xenobiotic reductase [EC:1.6.99.1] in the armillarioids (Supplementary Figure 5) it can also be speculated that they possess enhanced nitroglycerin-degrading potential. NADPH_2_ dehydrogenase might be involved in converting nitroglycerin to 1,2-dinitroglycerol. Pal and Christodoulatos (1995) used *P. chrysosporium* to study the nitroglycerin-degrading potential of fungi in a mixed batch reactor and observed that the fungus was highly efficient in degrading nitroglycerine and 2,4-dinitrotoluene. Our analysis shows that armillarioids have expanded nitroglycerine-degrading enzymes [EC:1.6.99.1]. Hence, armillariod species like *A. cepistipes* and *A. ostoyae* would be suitable candidates for *in vitro* screening experiments, besides other routinely used fungi like *P. chrysosporium*, *Penicillium corylophilum*, *A. fumigatus*, and *G. candidum*.

Maleyl acetate reductases are responsible for the breakdown of chloroaromatic compounds by directing maleyl acetate and its by-products to the 3-oxoadipate pathway (Seibert et al., 1993; Martins et al., 2015). BLAST analysis of armillarioid maleyl acetate dehydrogenase genes with the nr database of NCBI (National Centre for Biotechnology Information) indicated that their consensus sequence was 61% identical (E-value: 6e-145) with that of the maleyl acetate genes of *Pseudomonas* sp., showing that its presence in the fungal genomes may be the result of horizontal gene transfer.

*A. cepistipes* might be a promising candidate for arsenic biotransformation compared to other *Armillaria* and *Desarmillaria* species because of the high copy number of arsenite methyl transferases. *A. cepistipes* was previously found to be involved in removing heavy metals from the forest soil (Rigling et al., 2006), and also vanadium from media (Xu et al., 2019). Xu et al. (2019) studied the tolerance index of *A. cepistipes*, *Amanita muscaria*, *Xerocomus badius*, and *B. adusta* and compared them to one another. *A. cepistipes* showed acceptable tolerance towards sodium metavanadate (NaVO_3_) and vanadyl sulfate (VOSO_4_). However, the questions that remain to be answered are whether arsenite methyltransferases have a role in vanadium uptake and resistance and whether they also share a similar pathway for heavy metal accumulation.

Brunner et al. (2018) studied the plastic-degrading potential of *A. cepistipes* and *A. ostoyae* and various other fungal isolates. However, an experimental study of the possibility of fungi degrading polyurethane and polyethylene showed that none of the fungi could degrade the latter (Brunner et al., 2018). Therefore, it is clear from our study that the fungi we analyzed do not possess any genes with a potential role in polyethylene degradation.

Our comparative genomics approach provided a high-level overview of the mycoremediation potential of various fungal species that could efficiently degrade different kinds of pollutants. Although mycoremediation seems promising, various physical and chemical parameters play significant roles in determining the degradation efficiency of fungal strains when the operation is performed *in vitro* or *in situ*. For example, it was previously noted by Hefnawy et al. (2017) that a concentration of 0.05% direct blue dye inhibited the growth of *A. flavus* and *Penicillium canescens*, and they also found that concentrations beyond 0.01% were already toxic to the fungi, as was evidenced by decreased decolourization values. A similar consequence was observed for *P. chrysosporium* by Senthilkumar et al. (2014) when they increased the dye concentration; however, optimizing the media and introducing possible inducers such as lignin helped to significantly increase the tolerance. Factors such as temperature and pH were also observed to have crucial effects on biodegradation. For example, Hefnawy et al. (2017) found that *A. flavus* and *P. canescens* showed the best activity in the temperature and pH ranges of 30°C-35°C and 4-5, respectively. All these studies indicate that species with more significant biodegradation potential should be prioritized, and further intense research into optimizing the conditions of the preferred reaction pathways is crucial.

Bioremediation interventions primarily apply to the natural environment, but the application process can also be modeled and carried out in a controlled laboratory setting, where optimal conditions can be provided for the growth and activity of microorganisms or enzymes. Recent advancements in genetic and protein engineering have made laboratory procedures and bioengineering substantial in designing and developing more reliable solutions for mycoremediation. As a possible powerful alternative to employing native fungal cells, genetic engineering using the versatile CRISPR-Cas targeted genome editing technology could help to express the critically important genes or their edited versions in an environmentally or biotechnologically more suitable bacterial or fungal host (Jaiswal et al., 2019; Song et al., 2019; Singh et al., 2021). Importantly, the recently developed “HACKing” strategy may offer a highly reliable and efficient system for the co-expression of multiple genes in fungi used in environmental mycoremediation (Yue et al., 2023). Based on our current findings, testing the specialization and efficiency of the genetically diverse benzoate-monooxygenase homologs of the armillarioids in a heterologous system could be the first step toward a selective, targeted, and efficient biodegradation of certain aromatic, xenobiotic compounds in the environment.

## 5 Conclusion

Our study provides valuable insights into the importance of prior genome-wide analysis of the gene repertoires of various fungi potentially useable in biodegradation and biosorption techniques. We compared the potential biodegrading genes of armillarioid species to other ascomycetous and basidiomycetous fungi and identified the genes/enzymes that could be responsible for unique mycoremediation capabilities. The availability of such comparative data may offer novel and more reliable solutions in designing targeted mycoremediation applications to effectively treat specific environmental pollutants in natural environments. Comparative genomic analysis of 37 genomes showed that gene copy number patterns associated with biodegradation are generally conserved in both ascomycetous and basidiomycetous fungi, with notable exceptions at variable taxon levels. Results highlight the potential of white-rot fungi, particularly armillarioid species, in possible mycoremediation applications. Armillarioid species were found to have unique sets of genes, mainly involved in degrading aromatics, which clustered them separately from other white-rot species in a pPCA, suggesting that armillarioids have evolved unique strategies as well to degrade aromatics in their environment.

Our results demonstrate the benefit of using comparative genomics and transcriptomics data for the initial screening of fungal species and then identifying the most promising fungal candidates for a projected mycoremediation procedure. Future research could identify additional genes, explore further and elucidate the mechanisms underlying the relevant biodegradation pathways, leading to more sophisticated and efficient bioremediation procedures.

## Data Availability

The datasets presented in this study can be found in online repositories. The names of the repository/repositories and accession number(s) can be found in the article/Supplementary Material.
