# Corrigendum: The mycoremediation potential of the armillarioids: a comparative genomics analysis

**DOI:** 10.3389/fbioe.2024.1370053

**Published:** 2024-02-02

**Authors:** Simang Champramary, Boris Indic, Attila Szűcs, Chetna Tyagi, Omar Languar, K. M. Faridul Hasan, András Szekeres, Csaba Vágvölgyi, László Kredics, György Sipos

**Affiliations:** ^1^ Functional Genomics and Bioinformatics Group, Institute of Forest and Natural Resource Management, Faculty of Forestry, University of Sopron, Sopron, Hungary; ^2^ Department of Microbiology, Faculty of Science and Informatics, University of Szeged, Szeged, Hungary; ^3^ Fibre and Nanotechnology Program, Faculty of Wood Engineering and Creative Industries, University of Sopron, Sopron, Hungary

**Keywords:** mycoremediation, biodegradation, armillarioids, white-rot, phylogenetic principal component analysis, benzoate 4-monooxygenase

In the published article, there was an error in the **Funding** statement. The statement had to be completed, missing out on the funding of the publication fee. The original statement:

“This research was funded by Hungarian Government and the European Union within the frames of the Széchenyi 2020 Programme (GINOP-2.3.2-15-2016-00052).”

The correct Funding statement appears below.

