# Supplementary figures and images for: The mycoremediation potential of the armillarioids: a comparative genomics analysis
Source: Front Bioeng Biotechnol. 2023 Aug 17;11:1189640. doi: 10.3389/fbioe.2023.1189640 (PMC10470841; doi:10.3389/fbioe.2023.1189640)

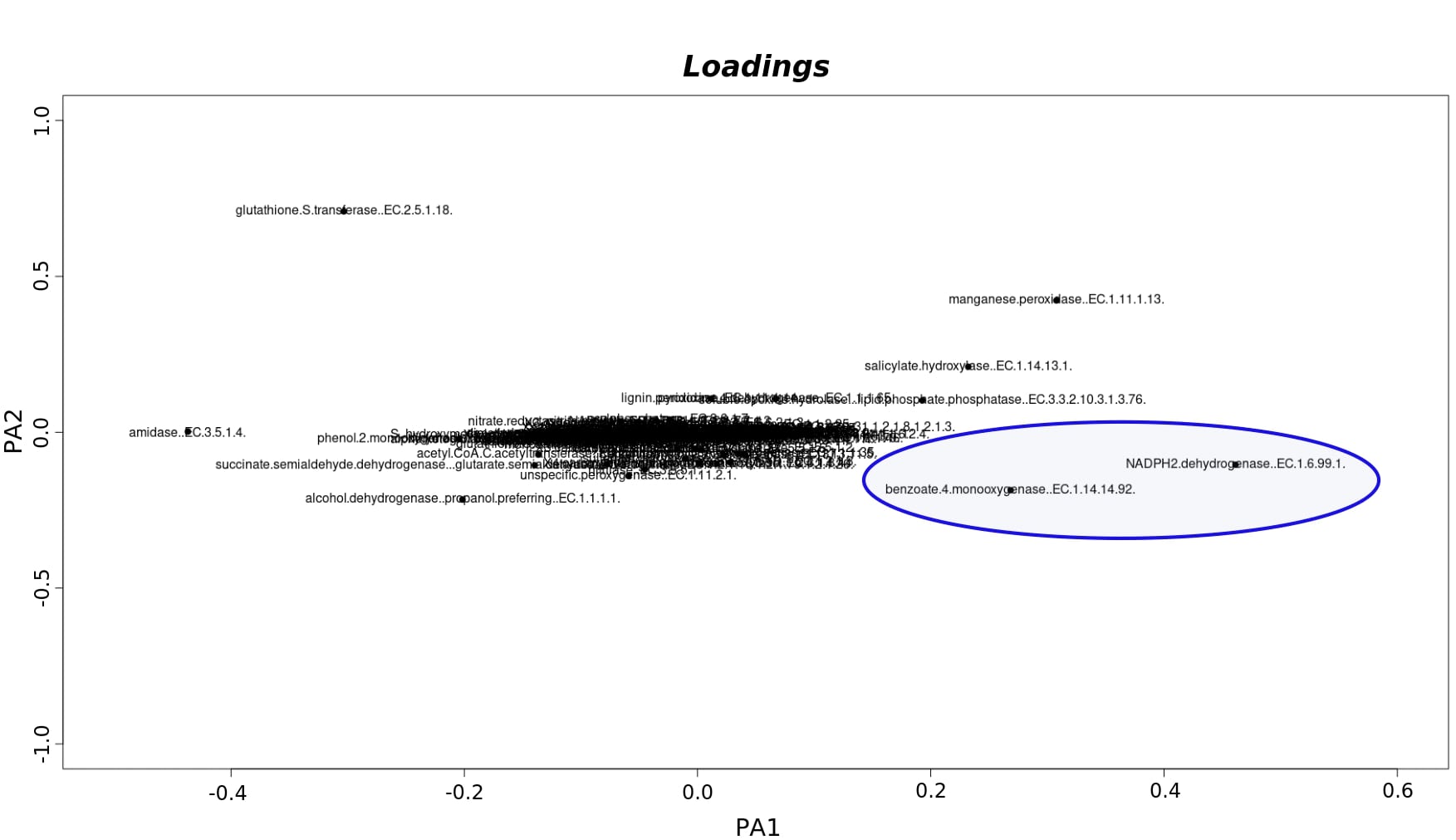

Supplement: Supplementary file 1 [file Image3.JPEG]

# Benzoate-4-monooxygenases

## Substrate binding sites

## Heme binding sites

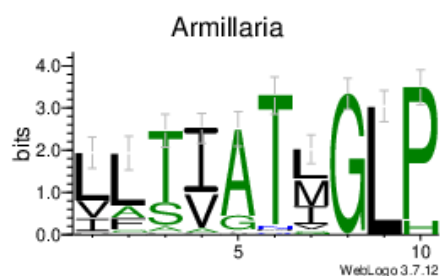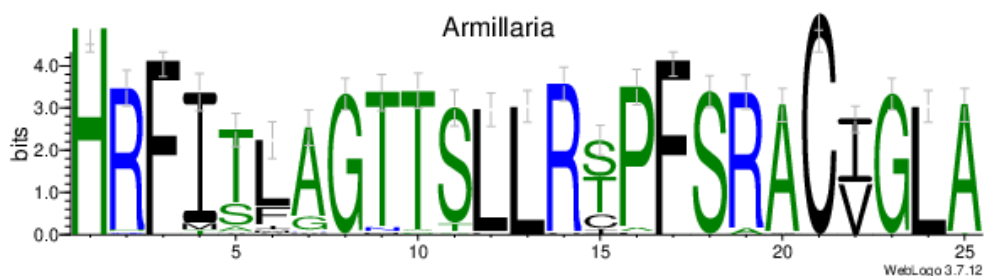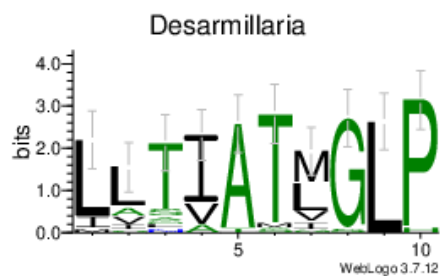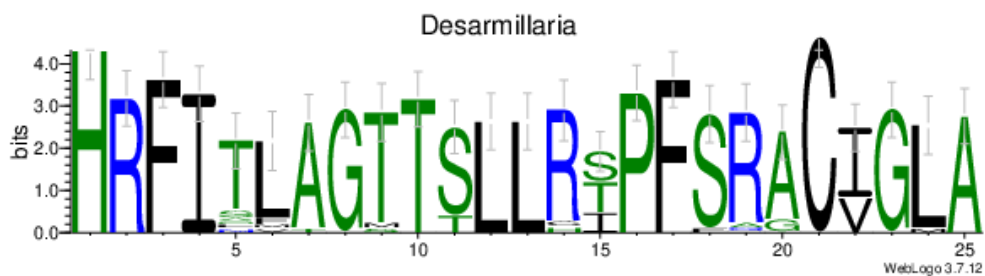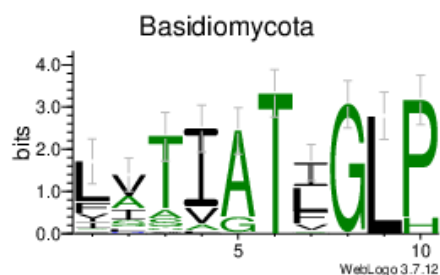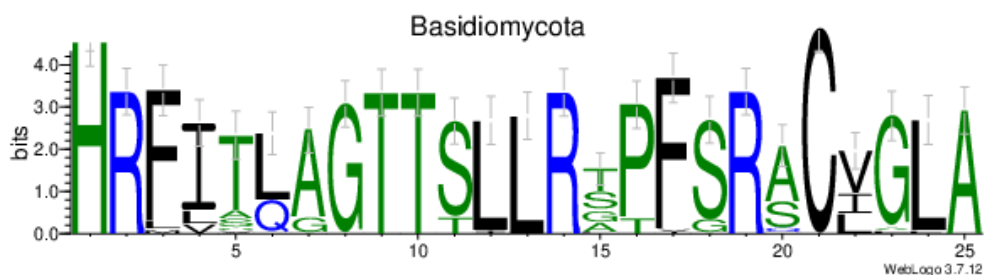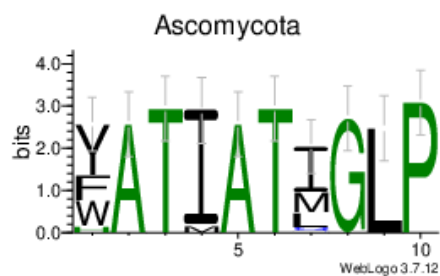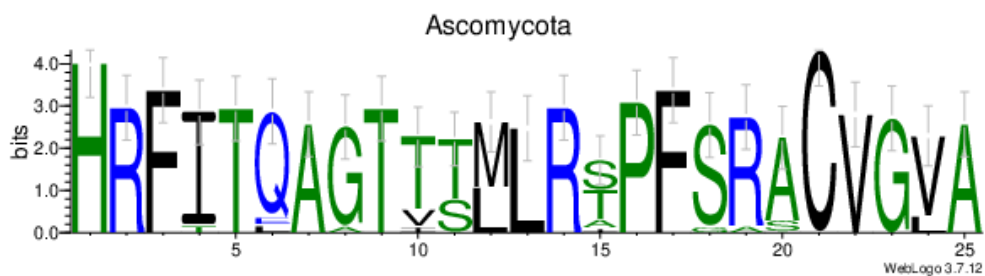

Supplement: Supplementary file 2 [file Image6.pdf]

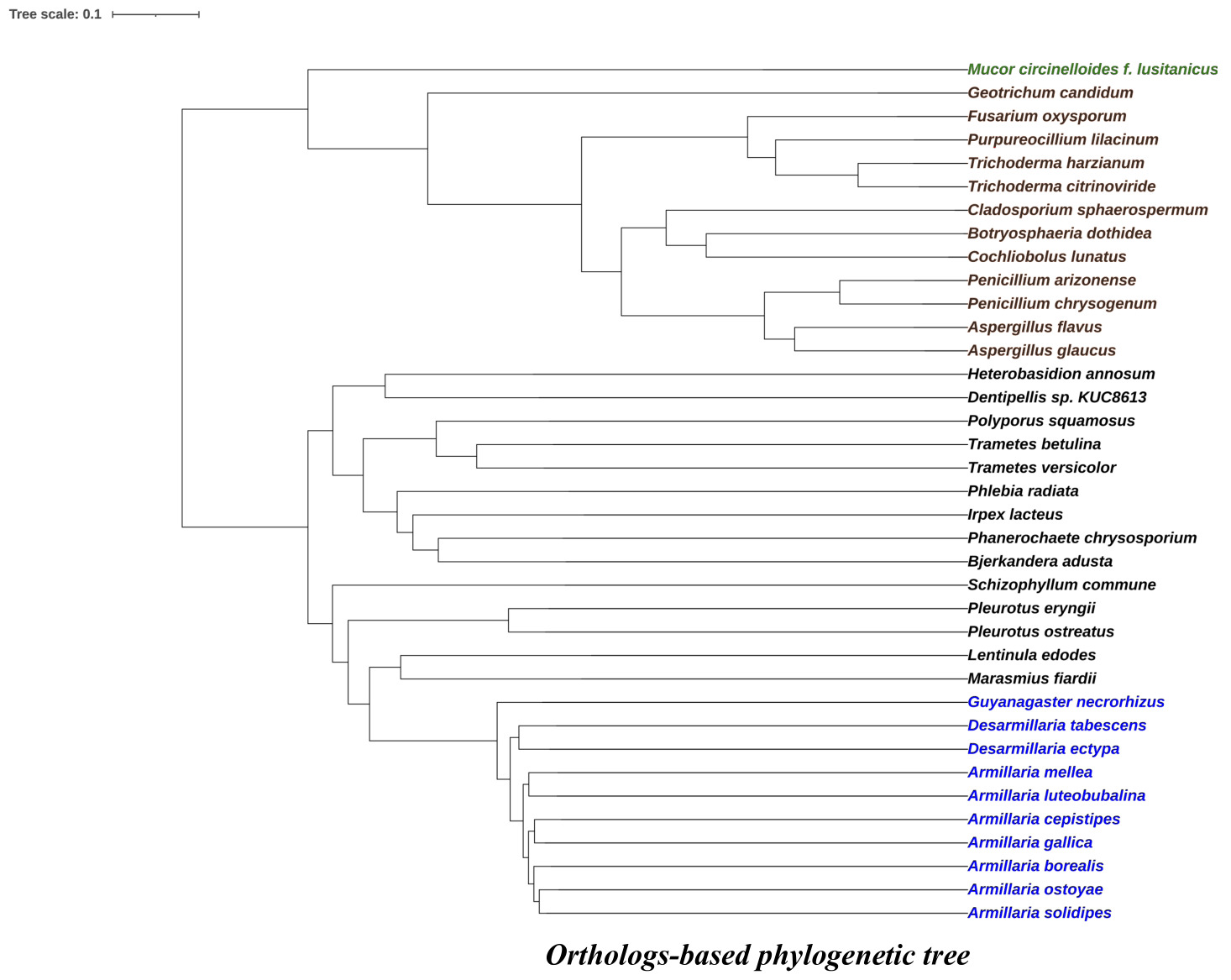

Supplement: Supplementary file 3 [file Image1.JPEG]

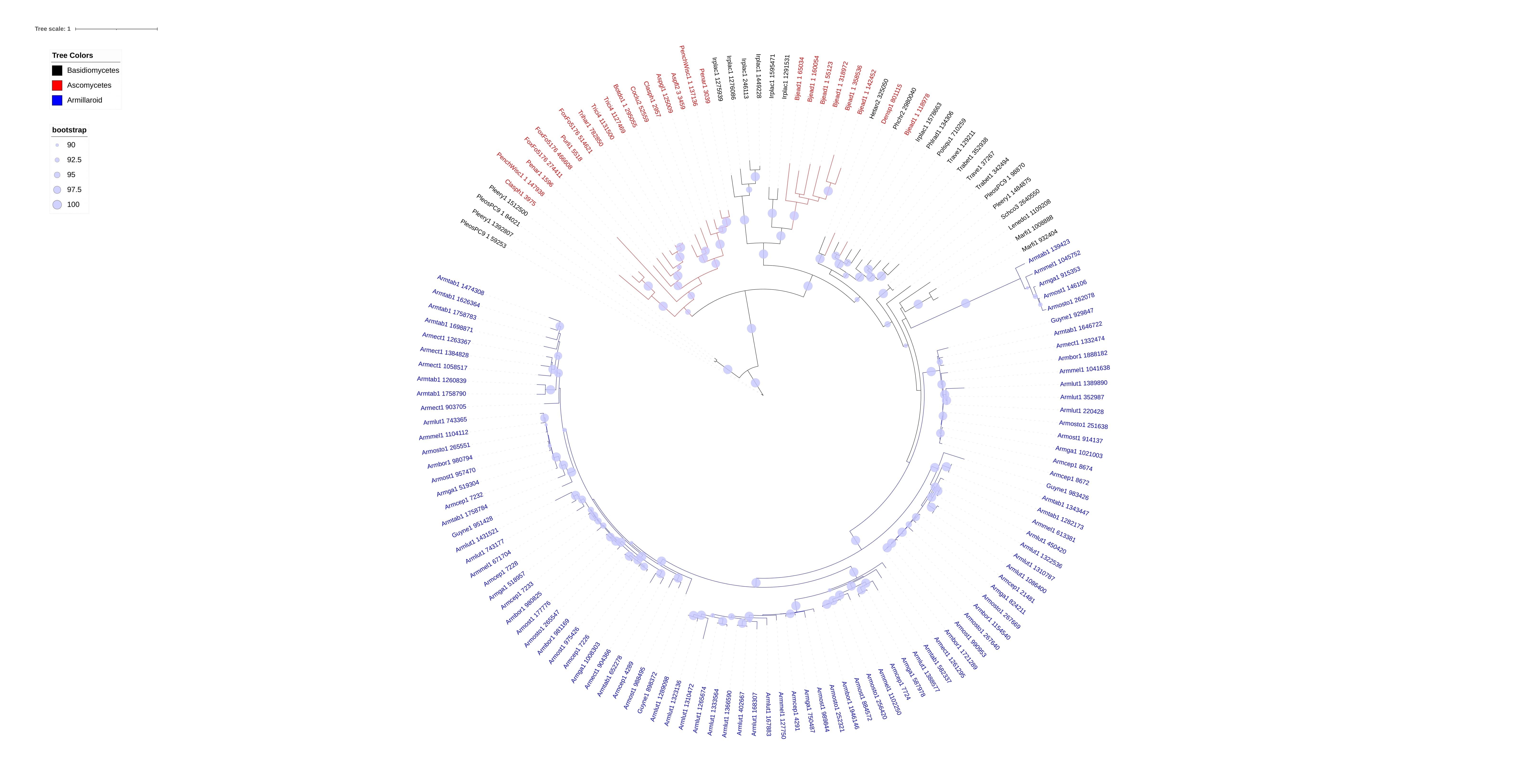

Supplement: Supplementary file 4 [file Image4.JPEG]

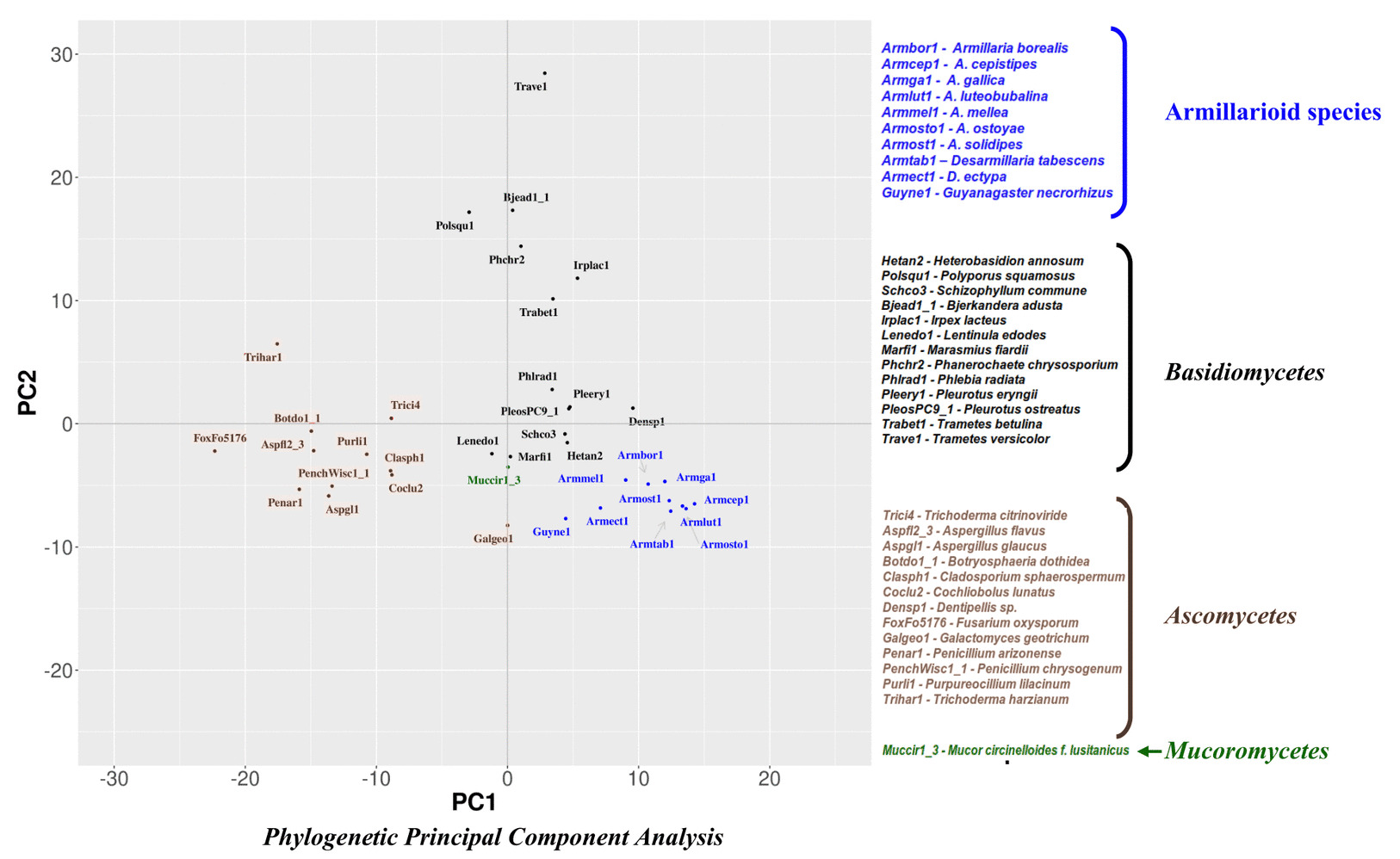

Supplement: Supplementary file 5 [file Image2.JPEG]

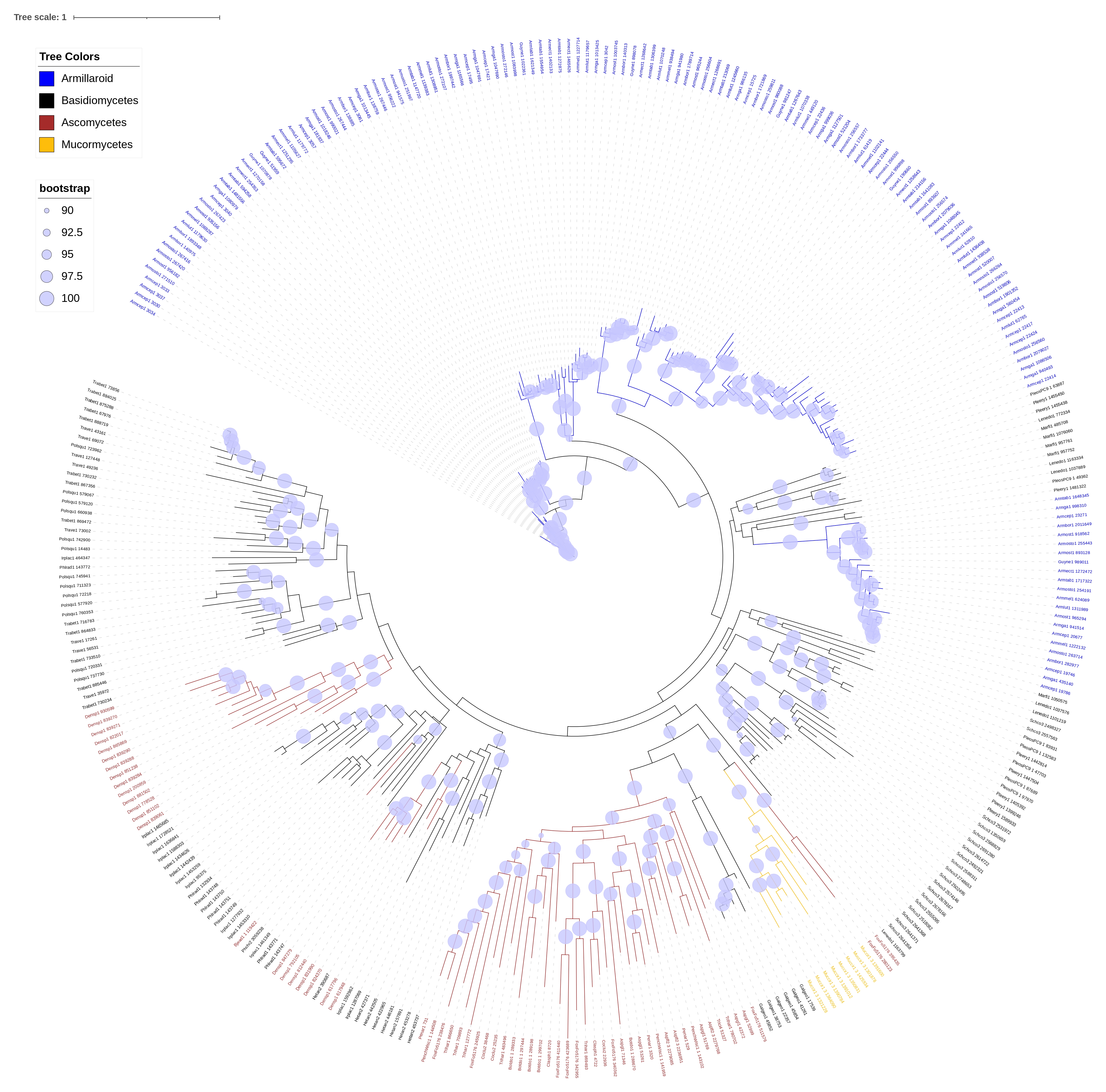

Supplement: Supplementary file 6 [file Image5.JPEG]
