# Supplementary material for: The mycoremediation potential of the armillarioids: a comparative genomics analysis
Source: Front Bioeng Biotechnol. 2023 Aug 17;11:1189640. doi: 10.3389/fbioe.2023.1189640 (PMC10470841; doi:10.3389/fbioe.2023.1189640)
Supplement: Supplementary file 8 [file DataSheet1.docx]

Supplementary Material

The mycoremediation potential of the Armillarioids: a comparative genomics analysis

**Simang Champramary^1,2^, Boris Indic^1^, Attila Szűcs^2^, Chetna Tyagi^2^, Omar Languar^1,^****^2^, K.M. Faridul Hasan^3^, András Szekeres^2^, Csaba Vágvölgyi^2^, László Kredics^2^ and György Sipos^1,^***

^1^Functional Genomics and Bioinformatics Group, Inst. of Forest and Natural Resource Management, Faculty of Forestry, University of Sopron, Bajcsy-Zsilinszky str. 4., H-9400 Sopron, Hungary

^2^Department of Microbiology, Faculty of Science and Informatics, University of Szeged, Közép fasor 52. H-6726 Szeged, Hungary

^3^Fibre and Nanotechnology Program, Faculty of Wood Engineering and Creative Industries, University of Sopron, Bajcsy-Zsilinszky str. 4., H-9400 Sopron, Hungary

*** Correspondence:**  sipos.gyorgy@uni-sopron.hu

# 1 Supplementary Data

**Supplementary File 1.** Excel file of data used in the comparative genomics and transcriptomics studies. The tab organism list consists of scientific names and fungal sources utilized in this study. The tab enzyme counts contain a matrix of individual enzymes in different fungal species and the enzyme class distribution tab denotes the enzyme class counts in all the fungal species. Finally, the last two tabs (*A. ostoyae* and *A. borealis*) refer to the gene expression data from the *in vitro* stem invasion assays.

# 2 Supplementary Figures

**Supplementary Figure 1.** Maximum likelihood phylogenetic tree of fungal species created using orthologous proteins.

**Supplementary Figure 2.** Phylogenetic principal components analysis based on the copy number of mycoremediation-related enzymes. Armillarioid species are denoted in blue, other basidiomycetes in black, and ascomycetes in brown. Mucoromycetes form the outgroup marked in green.

**Supplementary Figure 3.** Loadings plot of phylogenetic principal component analysis. The higher copy number of benzoate 4-monooxygenases and NADPH_2_ dehydrogenases circled were found higher in the armillarioid species and contributed to the separation of armillarioid species from the other fungi.

**Supplementary Figure 4.** Maximum likelihood phylogenetic tree showing the diversities of benzoate 4-monooxygenases.

**Supplementary Figure 5.** Maximum likelihood phylogenetic tree showing the diversities of NADPH_2_ dehydrogenases.

**Supplementary Figure 6.** Sequence alignments of substrate and heme binding sites of the benzoate 4-monooxygenases.
